# Maternal iron-and-folic-acid supplementation and its association with low-birth weight and neonatal mortality in India

**DOI:** 10.1017/S1368980021004572

**Published:** 2022-03

**Authors:** Rajesh Kumar Rai, Jan-Walter De Neve, Pascal Geldsetzer, Sebastian Vollmer

**Affiliations:** 1 Society for Health and Demographic Surveillance, Suri, WB 731101, India; 2 Department of Global Health and Population, Harvard T H Chan School of Public Health, Boston, MA, USA; 3 Department of Economics, University of Goettingen, Goettingen, Germany; 4 Centre for Modern Indian Studies, University of Goettingen, Goettingen, Germany; 5 Heidelberg Institute of Global Health, Faculty of Medicine and University Hospital, University of Heidelberg, Heidelberg, Germany; 6 Division of Primary Care and Population Health, Department of Medicine, Stanford University, California, USA; 7 Centre for Poverty, Equity and Growth, University of Goettingen, Goettingen, Germany

**Keywords:** Anaemia, Fe-deficiency anaemia, Iron-and-folic-acid, Micronutrients, India

## Abstract

**Objective:**

The current study assessed intake of iron-and-folic-acid (IFA) tablet/syrup (grouped into none, < 100 d of IFA consumption or < 100 IFA and ≥ 100 d of IFA consumption or ≥ 100 IFA) among prospective mothers and its association with various stages of low-birth weight (ELBW, extremely low-birth weight; VLBW, very low-birth weight and LBW, low-birth weight) and neonatal mortality (death during day 0–1, 2–6, 7–27 and 0–27) in India.

**Design:**

The cross-sectional, nationally representative, 2015–2016 National Family Health Survey (NFHS-4) data were used. Weighted descriptive analysis and multiple binary logistic regression modelling were used.

**Setting:**

NFHS-4 covered 640 districts from thirty-seven states and union territories of India.

**Participants:**

A total of 120 374 and 143 675 index children aged 0–59 months were included to analyse LBW and neonatal mortality, respectively.

**Results:**

Overall, 30·7 % mothers consumed ≥ 100 IFA in 2015–2016, and this estimate ranged from 0·0 % in Zunheboto district of Nagaland state to 89·5 % in Mahe district of Puducherry of India. Multiple regression analysis revealed that children of mothers who consumed ≥ 100 IFA had lower odds of ELBW, VLBW, LBW and neonatal mortality during day 0–1, as compared with mothers who did not buy/receive any IFA. Consumption of IFA (< 100 IFA and ≥ 100 IFA) had a protective association with neonatal death during day 7–27 and 0–27. Consumption of IFA was not associated with neonatal death during day 2–6.

**Conclusions:**

While ≥ 100 IFA consumption during pregnancy was found to be associated with preventing select types of LBW and neonatal mortality, a large variation in coverage of ≥ 100 IFA consumption across 640 districts is concerning.

India has the largest anaemic population in the world^(1)^. Anaemia is defined as concentration of Hb in the blood below an established threshold^(2)^. Fe-deficiency anaemia (IDA) is the most common cause of anaemia, but other nutritional deficiencies (including folate, vitamin B_12_ and vitamin A), acute and chronic inflammation, parasitic infections and hereditary or acquired disorders that affect Hb synthesis, red blood cell production or red blood cell survival can also cause anaemia^(1–3)^. According to the 2017 Global Burden of Disease study^(4)^, IDA was the leading cause of years lived with disability in India, resulting in an unprecedented loss to the country’s productivity^(5,6)^. IDA disproportionately affects women, especially pregnant women^(7,8)^. In 2018, an estimated 181·3 million women were found to be anaemic (95 % CI: 171·4, 190·2) in India, of which an estimated 103·4 million (95 % CI: 94·2, 112·7) were moderately or severely anaemic^(9)^. As compared with a non-anaemic pregnant woman, an anaemic pregnant woman has higher likelihood of pregnancy complications, experiencing adverse pregnancy and poor birth outcomes such as preterm birth (PTB), intrauterine growth restriction, stillbirth, low-birth weight (LBW) and neonatal deaths^(1,7–11)^.

To combat the IDA burden among its most vulnerable populations – children, adolescents, pregnant and lactating mothers and women – the Indian government has undertaken various initiatives, starting from the National Nutritional Anaemia Prophylaxis Programme launched in 1970, followed by the 1991 National Nutritional Anaemia Control Programme, the 2012 Weekly Iron-and-Folic-Acid Supplementation Programme and the National Iron Plus Initiative launched in 2013. Despite these multiple initiatives, over 50 % of all women (including pregnant and non-pregnant women) aged 15–49 years were found to be anaemic in 2015–2016^(12)^. Learning from the failure of anaemia reduction initiatives ^(13–17)^, a programme called the Prime Minister’s Overarching Scheme for Holistic Nourishment (POSHAN) *Abhiyaan* (or National Nutrition Mission) was set up in 2018 to address nutrition issues under the oversight of the Ministry of Women & Child Development, Government of India. The POSHAN *Abhiyaan* pledged for an Anaemia *Mukt Bharat* (Anaemia Free India) and targeted reduction in anaemia among adolescent girls and women aged 15–49 years at the rate of three percent per annum^(18)^.

Antenatal iron-and-folic-acid (IFA) supplementation is a cost-effective public health intervention to avert poor pregnancy outcomes that may occur due to anaemia during pregnancy^(19,20)^. The National Iron Plus Initiative guideline recommends that pregnant women should consume a dose of 100 mg of elemental Fe and 500 mcg of folic acid daily for at least 100 d (≥ 100 IFA), starting after the first trimester, at 14–16 weeks of gestation^(1)^. The supplementation of elemental Fe is expected to correct Fe deficiency and IDA among pregnant women, which would in turn help reduce the chances of adverse birth outcomes and strengthen the health of new-borns^(1,21)^. While execution and effectiveness of existing public programmes have been heavily criticised, the uptake of IFA among pregnant women has been sub-optimal^(13)^.

The empirical evidence on the effect of IFA supplementation to pregnant mothers on LBW and survival status of their children in India is at a premature stage. Existing studies on this issue are either outdated^(22–24)^ or focus on specific administrative regions of India^(10)^ and small sample studies are prone to low external validity. Against this knowledge gap, using cross-sectional, nationally representative data from India, we assessed whether maternal consumption of IFA (categorised into three groups: none, < 100 IFA and ≥ 100 IFA) during their last pregnancy were associated with selected child health indicators – extremely low-birth weight (ELBW), very low-birth weight (VLBW), low-birth weight (LBW) and neonatal mortality (death during days 0–27) including death during day 0–1, day 2–6 and day 7–27. To date, no study has assessed the association between IFA and various stages of LBW and neonatal mortality in India. Findings of the current study could be helpful in exploring the need for targeted IFA intervention in mitigating overall LBW and neonatal deaths in India. As an add-on analysis, we also estimated the change (between 2005–2006 and 2015–2016) in prevalence of ≥ 100 IFA consumption across states in India, whereas coverage of ≥ 100 IFA uptake was analysed for 2015–2016 in 640 districts.

## Methods

### Data set

The 2015–2016 National Family Health Survey (NFHS-4) data were used to attain the study objectives^(12)^. NFHS-4 is a cross-sectional and nationally representative survey conducted under the stewardship of the Ministry of Health and Family Welfare, Government of India. NFHS-4 covered 640 districts spread across thirty-seven states and union territories of India. The 2011 Census of India sampling frame was used to draw the sample for both rural and urban areas using two-stage stratified random sampling. Villages in rural areas and census enumeration blocks (CEB) in urban areas served as the primary sampling unit (PSU) or clusters. With household response rates above 97 %, a total of 601 509 households were selected, consisting of 699 686 women and 112 122 men in NFHS-4. Details of the NFHS-4 sampling procedures are available in its published report^(12)^. NFHS-4 data are available in the public domain with all participant identifiers removed. Prior to conducting the NFHS-4, ethical approval was obtained by the nodal agency – International Institute for Population Sciences, Mumbai – from the independent ethics review committee constituted by the Ministry of Health and Family Welfare, Government of India.

In NFHS-4, a total of 190 797 mothers were asked about their IFA consumption for their index pregnancy. To analyse LBW, a total of 120 374 children were found eligible, whereas the denominator for analysing neonatal mortality was 143 675 children. As the exclusion of survey participants may lead to sample selection bias, the sample included in the analysis was compared with the sample excluded from the analysis for age and sex of children. NFHS-4 records information on birth weight for children born in the 5 years preceding the survey date, whereas age at death was recorded for children ever born. However, the current study included record of birth weight and/or child mortality only for index children which helped minimise recall errors. The prevalence of ≥ 100 IFA in 2015–2016 was compared with ≥ 100 IFA in 2005–2006 across twenty-nine states and union territories of India to understand the change in coverage of ≥ 100 IFA over the last decade. The 2005–2006 National Family Health Survey (NFHS-3) data^(25)^ were compared with NFHS-4, and by virtue of their sampling design, estimates from both rounds of NFHS are comparable^(26)^. In addition, the coverage of ≥ 100 IFA intake in NFHS-4 was analysed for 640 districts spread across thirty-seven states and union territories of India.

### Outcome events

**Fig. 1:**
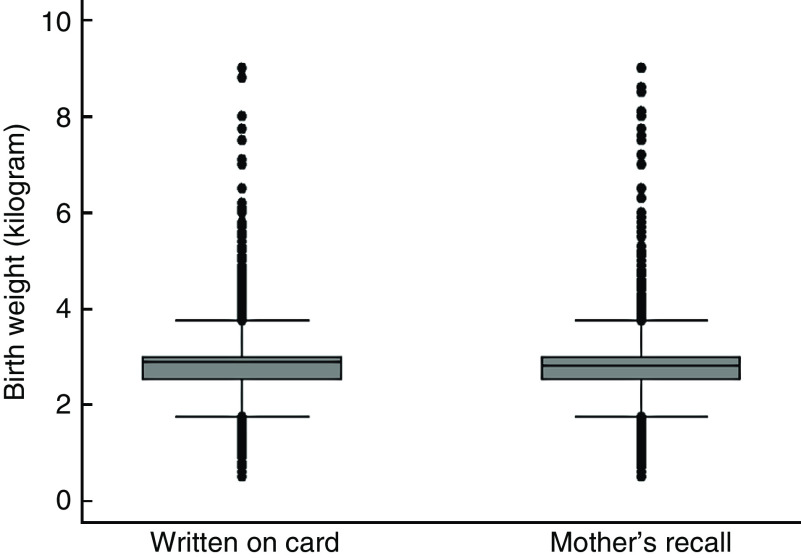
Box plot showing the distribution of birth weight datapoints recorded from health card and mother’s recall

Two outcome events were analysed – LBW and neonatal mortality and their sub-categories. According to WHO, LBW is defined as weight at birth of <2·5 kg or 5·5 pounds^(27)^. For the current study, children’s birth weight was categorised into three groups: low-birth weight (LBW) with weight of < 2·5 kg, VLBW with weight of < 1·5 kg and ELBW with weight of < 1·0 kg^(27)^. In NFHS-4, women were asked if the children born to them were weighed at birth, and if the response was affirmative, the weight at birth was recorded in kg. It was advised to record the birth weight from their government issued health card (i.e. a written record of the birth weight on a government issued document, such as the vaccination card, the antenatal card or the birth certificate), if available, otherwise reporting of birth weight was based on the mother’s recall from memory. Of 120 374 children included for analysing LBW, birth weight of 55 227 (unweighted) children (42·8 %) were based on mother’s recall (calculated by authors, from NFHS-4 data). The distribution of birth weight datapoints from health card and mother’s recall is presented in Fig. 1. The mean birth weight collected from health cards was 2·82 kg (95 % CI: 2·81, 2·83), whereas the mean birth weight from mother’s recall was estimated to be 2·80 kg (95 % CI: 2·79, 2·81), and an independent group *t* test indicated that the mean of birth weight between two groups (from health card and mother’s recall) was different with a two-tailed *P*-value of < 0·001. In the women’s questionnaire, the birth and death history of children ever born to them was recorded. If the child was reported to be dead, a further question was posed on age at which the child died. Age at death was recorded in days if the child died within the first month of life, in months if the child died between one month and the second birthday, or otherwise in years. In the current study, death of children during the first 28 d of life (0–27 d) is defined as neonatal mortality. Neonatal mortality was further investigated by age at death: day 0–1, day 2–6 and day 7–27. This categorisation of age of death is critical as neonatal death varies greatly with days^(28)^, and analysing the role of IFA on various categories of neonatal deaths would help understand if IFA consumption is associated with neonatal death of a particular age-group.

### Primary-independent variable and covariates

While IFA consumption was used as a primary variable, a range of covariables were considered as potential confounders. In NFHS-4, women aged 15–49 years were asked – whether they were given or if they had bought any IFA tablets or equivalent syrup during their last pregnancy in the 5 years preceding the survey date. If the response was affirmative, they were asked about the number of days they took the tablet or syrup, and if the answer was non-numeric, they were probed about approximate number of days (for example, by asking how many months pregnant she was when she began taking the tablets and whether she took the tablets every day after that). Although asking approximate number of days of IFA consumption may lead to recall errors, this information is deemed useful for understanding overall coverage of IFA consumption and for informing public health policy in India^(13,24,29)^. The NFHS-4 does not collect information about the proportion of women who required probing to obtain information on number of days of IFA consumption during their pregnancy. Interviewers were asked to show sample IFA tablets or syrup to the respondents while asking the questions on IFA to minimise recall errors. Inclusion of information on IFA consumption for index birth refers to a birth in 2011 or later for NFHS-4. Using the information on IFA consumption, the primary variable of interest was computed into three categories: none, < 100 d of IFA consumption (< 100 IFA) and ≥ 100 d of IFA consumption (≥ 100 IFA). The category ‘none’ represents the group of women who did not receive or buy any IFA during their last pregnancy. Consumption of < 100 IFA include women who received or bought IFA but did not consume any of it, and their proportion is negligible (< 0·5 %). A maximum of 300 d of IFA tablets or equivalent syrup consumption was recorded in NFHS-4. On average, the gestation for term pregnancy lasts 40 weeks (280 d); therefore, women with term pregnancy are expected to consume a maximum of 280 IFA. However, reporting of consumption of > 280 IFA (nearly 3·1 % of all mothers) is indicative of mothers who had post-term pregnancies^(30)^.

The conceptualisation of association between IFA consumption (exposure variable) and child health indicators of LBW and neonatal mortality (outcome variables) was guided by a directed acyclic graph^(31,32)^, available in the online supplement (see online supplementary material, Supplemental Fig. S1). Although the current study does not establish the causal link between IFA intake and selected child health indicators, a directed acyclic graph may help establish the pathways between exposure and outcome variables of interest by identifying potential confounders (confounding is the bias of the estimated effect of an exposure on an outcome due to the presence of a common cause of the exposure and the outcome), mediator (a variable that lies ‘between’ the exposure and the outcome) and collider (a variable directly affected by two or more other variables in the causal diagram), guided by existing literature on the issue. Based on a directed acyclic graph and depending on the available information in NFHS-4 data set, a range of covariables were identified as potential confounders. Potential confounders included current age group of mother (15–19, 20–29, 30–39 and ≥ 40), mother’s age at marriage (< 18 and ≥ 18), education of mother (none or incomplete primary, primary or incomplete secondary and secondary or higher), sex of child (male and female), child birth order (1, 2, 3, 4 and ≥ 5), place of residence (urban and rural), social group (Others, Scheduled Castes, Scheduled Tribes and Other Backward Classes), religion (Hinduism, Islam, Christianity and others), economic group (poorest, poorer, middle, richer and richest), state of residence (‘non-high focus’ and ‘high focus’ defined below), antenatal care (ANC) and delivery care are clubbed under number of ANC visits (< 4 and ≥ 4), received supplementary food from *Anganwadi* centre (yes and no), mother’s blood sample taken during ANC visit (yes and no) and institutional delivery (yes and no), where maternal nutrition was based on measurement of BMI or BMI (underweight, optimum and overweight including obesity). In addition, variables on sources of birth weight data (e.g. written health card and mother’s recall) were also identified as a potential confounder for the birth weight analysis.

Primary education refers to grades 1 to 8, while secondary education refers to grades 9 to 10. Of social group, as per the Constitution of India^(33)^, Scheduled Tribes, Scheduled Castes and (so called) Other Backward Classes are historically, socially and economically disadvantaged populations compared with the rest of the population (labelled as Others). NFHS-4 includes a wealth index variable, calculated using assets and durables owned by the household, which included ownership of consumable items and dwelling characteristics. Individuals were ranked based on their household scores and divided into different quintiles, each representing 20 % of the score, between 1 (poorest) and 5 (richest)^(34)^. Because of their high fertility and high mortality indicators, the following nine states are regarded as high focus states: Bihar, Chhattisgarh, Jharkhand, Madhya Pradesh, Odisha, Rajasthan, Uttarakhand, Uttar Pradesh and Assam^(35)^. Under the 2013 National Food Security Act, pregnant women are entitled to receive cooked or take-home ration during their pregnancy from the *Anganwadi* centre (meaning, ‘courtyard shelter’)^(36)^. According to the WHO, a BMI of < 18·5 kg/m^2^ is considered a measure of underweight, 18·5–22·99 kg/m^2^ as optimum weight and ≥ 23 kg/m^2^ is labelled as the measure of overweight including obesity for Asian populations^(37)^.

The objective of IFA supplementation during pregnancy is to correct Fe deficiency and IDA that depends on bioavailability, gut integrity, Fe stores and infection^(38)^. Anaemia level during pregnancy can act as a potential mediator in establishing the linkages between IFA consumption and child health indicators. But NFHS-4 does not collect information on Hb level during pregnancy retrospectively, instead NFHS-4 measures anaemia level among women at the time of survey that cannot be used and the proxy measure of anaemia during their last pregnancy.

### Statistical analysis

A combination of descriptive statistics and multiple regression analyses was used. In addition, an analysis of changes in the coverage of ≥ 100 IFA intake (between 2005–2006 and 2015–2016) and prevalence of ≥ 100 IFA consumption across 640 districts of India were undertaken. Bivariate analysis was run to understand the proportional difference of outcome events – ELBW, VLBW, LBW and neonatal death (day 0–27) stratified by days of death (day 0–1, day 2–6 and day 7–27) by IFA consumption and other potential confounders. Prior to running bivariate analysis, a χ^2^ test was run to understand if the distribution of children’s age and sex differ between the sample included in and the sample excluded from the analysis. Multiple logistic regression analyses were conducted for all outcome variables coded in binary terms (1 and 0), where occurrence of the outcome event was coded as ‘1’ and its absence was coded as ‘0’. For each outcome, the model included the primary variable of interest – IFA intake (categorised into three groups – none, < 100 IFA and ≥ 100 IFA), variables representing socio-economic characteristics, variables representing ANC and delivery care and maternal BMI. For all the outcomes on birth weight, one additional variable representing source of birth weight was adjusted as birth weight reporting could differ between the health card and maternal recall^(22)^. Also, while running the regression models for LBW, sex of the child is not a confounder in any of the models.

Recording of birth weight through mother’s recall is likely to have digit preference, often in multiples of 500 g^(22)^ which leads to heaping^(39)^. To check the sensitivity of it, an alternate analysis with alternate definition of ELBW of ≤ 1·0 kg, VLBW ≤ 1·5 kg and LBW of ≤ 2·5 kg was done. Appropriate sample weighting provided with the NFHS data set was used for running descriptive analysis and ‘svy’ suite available to adjust sample weighting with the statistical software Stata, version 14^(40)^ was used. While weighted descriptive analysis was run, unweighted multiple binary logistic regression models^(41)^ were developed to understand the association between the primary variable and various stages of LBW and neonatal mortality.

## Results

**Fig. 2:**
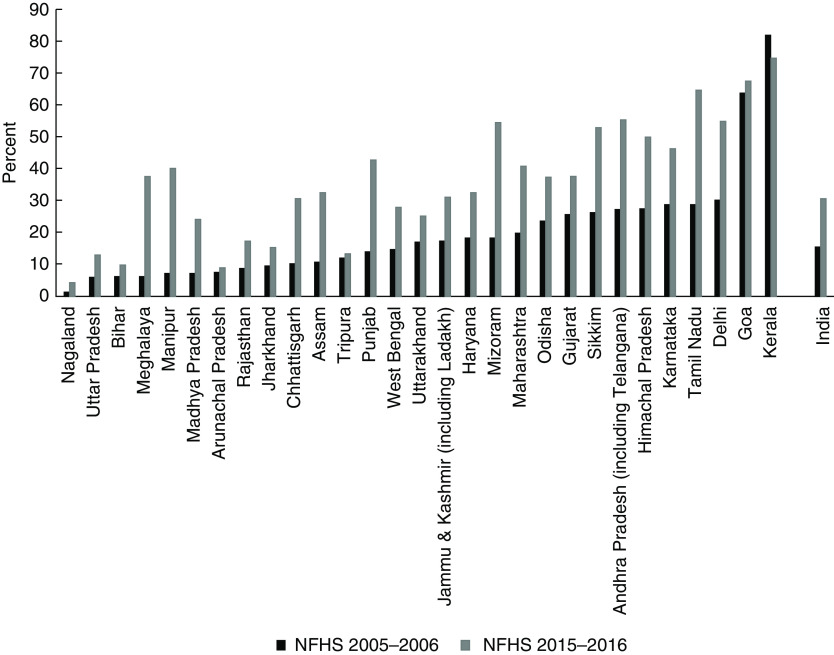
Change in prevalence (%) of ≥ 100 iron-and-folic-acid receipt between 2005–2006 and 2015–2016, in 29 states and union territories of India. During survey period of the 2005–2006 National Family Health Survey, Ladakh was part of Jammu & Kashmir; and during survey period of the 2005–2006 National Family Health Survey, Telangana was part of Andhra Pradesh. NFHS, National Family Health Survey

The sample included in the analysis was checked for sample selection bias, and the distribution of child age and sex was not different between the sample included in and the sample excluded from the analysis (data not shown separately). An analysis of changing coverage of ≥ 100 IFA intake by states (see online supplementary material, Supplemental Table S1, and Fig. 2) between 2005–2006 and 2015–2016 was conducted, followed by extent of coverage of ≥100 IFA intake across 640 districts in India (see online supplementary material, Supplemental Table S2). Overall, the coverage of ≥ 100 IFA intake doubled between 2005–2006 (15·6 %) and 2015–2016 (30·7 %). During the same time, the state of Mizoram gained 36·2 percentage points in ≥ 100 IFA intake, whereas the state of Kerala, with 74·8 % coverage in 2015–2016, saw a 6·9 percentage point reduction of ≥ 100 IFA intake. District-wise coverage of ≥ 100 IFA in 2015–2016 ranged from 0·0 % in Zunheboto districts of Nagaland to 89·5 % in Mahe district of Puducherry. Among 640 districts in India, the state of Nagaland has five districts (Zunheboto, Longleng, Mon, Phek and Kiphire) with the lowest coverage of ≥ 100 IFA intake, followed by three districts in Arunachal Pradesh (West Siang, Upper Subansiri and East Kameng).

Prevalence with 95 % CI of ELBW, VLBW and LBW and prevalence of timing of neonatal mortality (day 0–1, day 2–6 and day 7–27) and neonatal mortality (day 0–27) by select background characteristics are presented in Table 1. Overall, 0·11 % (CI: 0·09, 0·14), 1·14 % (CI: 1·06, 1·23) and 17·0 % (CI: 16·7, 17·3) children had ELBW, VLBW and LBW, respectively. Prevalence of neonatal mortality is 1·69 % (CI: 1·61, 1·78), with 0·96 % (CI: 0·90, 1·02) reporting a death during day 0–1.

**Table 1:** Prevalence of extremely low-birth weight, very low-birth weight and low-birth weight and prevalence of timing of neonatal mortality (day 0–1, day 2–6 and day 7–27) and neonatal mortality (day 0–27) by primary variable and covariables

		Extremely low-birth weight		Very low-birth weight		Low-birth weight			Neonatal mortality (day 0–1)		Neonatal mortality (day 2–6)		Neonatal mortality (day 7–27)		Neonatal mortality, (day 0–27)	
	*n*	%	95 % CI	%	95 % CI	%	95 % CI	*n*	%	95 % CI	%	95 % CI	%	95 % CI	%	95 % CI
IFA consumption																
No IFA	16 751	0·17	0·11, 0·26	1·58	1·36, 1·85	19·1	18·3, 19·9	24 432	1·37	1·20, 1·56	0·61	0·51, 0·74	0·49	0·39, 0·61	2·47	2·24, 2·72
< 100 IFA	60 141	0·11	0·08, 0·15	1·21	1·10, 1·33	17·9	17·5, 18·4	72 651	1·01	0·92, 1·10	0·50	0·44, 0·57	0·29	0·24, 0·35	1·80	1·68, 1·92
≥ 100 IFA	43 482	0·09	0·05, 0·15	0·90	0·78, 1·05	15·1	14·6, 15·6	46 592	0·69	0·60, 0·80	0·33	0·27, 0·41	0·14	0·11, 0·18	1·17	1·05, 1·31
Current age-group of mother																
15–19	3764	0·11	0·04, 0·33	1·73	1·28, 2·34	20·8	19·0, 22·6	4364	1·40	1·06, 1·83	1·18	0·81, 1·73	0·65	0·43, 0·98	3·23	2·64, 3·94
20–29	83 556	0·10	0·07, 0·13	1·12	1·03, 1·22	16·9	16·6, 17·3	98 155	0·92	0·85, 1·00	0·43	0·38, 0·49	0·24	0·20, 0·28	1·60	1·50, 1·70
30–39	30 650	0·14	0·09, 0·23	1·11	0·96, 1·30	16·7	16·1, 17·3	37 755	0·95	0·83, 1·09	0·43	0·36, 0·52	0·30	0·24, 0·37	1·68	1·52, 1·85
≥ 40	2404	0·14	0·02, 1·00	1·40	0·89, 2·20	17·1	15·0, 19·5	3401	1·56	1·12, 2·17	0·56	0·33, 0·95	0·51	0·25, 1·04	2·64	2·03, 3·42
Mother’s age at first birth																
< 18	12 259	0·13	0·07, 0·26	1·19	0·96, 1·47	18·8	17·8, 19·8	15 728	1·03	0·85, 1·26	0·51	0·38, 0·68	0·26	0·19, 0·36	1·80	1·55, 2·09
≥ 18	108 115	0·11	0·08, 0·14	1·14	1·06, 1·23	16·8	16·5, 17·1	127 947	0·95	0·88, 1·01	0·45	0·41, 0·50	0·27	0·24, 0·31	1·67	1·59, 1·77
Education of mother																
No or incomplete primary	30 580	0·13	0·09, 0·20	1·48	1·31, 1·67	19·4	18·8, 20·0	43 064	1·40	1·27, 1·55	0·62	0·54, 0·72	0·39	0·32, 0·47	2·41	2·24, 2·60
Primary or incomplete secondary	59 973	0·11	0·08, 0·15	1·15	1·04, 1·27	17·5	17·1, 18·0	68 896	0·89	0·80, 0·98	0·44	0·38, 0·51	0·27	0·22, 0·32	1·59	1·48, 1·72
Secondary or higher	29 821	0·09	0·04, 0·17	0·83	0·69, 0·99	13·8	13·3, 14·4	31 715	0·57	0·48, 0·67	0·30	0·23, 0·40	0·15	0·11, 0·20	1·02	0·89, 1·16
Sex of child																
Male	na	na		na		na		78 140	1·01	0·92, 1·10	0·48	0·42, 0·54	0·27	0·22, 0·32	1·75	1·64, 1·87
Female	na	na		na		na		65 535	0·90	0·81, 0·99	0·44	0·38, 0·51	0·28	0·23, 0·34	1·62	1·50, 1·74
Birth order																
1	45 200	0·08	0·06, 0·12	1·21	1·08, 1·37	17·7	17·2, 18·2	49 993	0·96	0·86, 1·06	0·53	0·45, 0·62	0·24	0·19, 0·29	1·72	1·59, 1·87
2	42 394	0·11	0·07, 0·18	0·94	0·83, 1·07	15·9	15·4, 16·4	48 925	0·69	0·61, 0·79	0·34	0·28, 0·40	0·23	0·17, 0·30	1·25	1·13, 1·38
3	18 856	0·13	0·08, 0·23	1·15	0·96, 1·38	16·9	16·2, 17·7	23 997	1·10	0·93, 1·30	0·38	0·30, 0·48	0·31	0·23, 0·42	1·79	1·58, 2·03
4	7917	0·16	0·08, 0·32	1·72	1·38, 2·13	18·4	17·3, 19·6	11 126	1·45	1·19, 1·77	0·71	0·54, 0·94	0·34	0·23, 0·50	2·50	2·16, 2·90
≥ 5	6007	0·15	0·06, 0·37	1·52	1·18, 1·95	19·0	17·7, 20·3	9634	1·66	1·38, 2·01	0·72	0·54, 0·97	0·61	0·44, 0·85	3·00	2·60, 3·46
Place of residence																
Urban	35 312	0·10	0·06, 0·16	1·10	0·96, 1·27	16·0	15·4, 16·6	39 157	0·72	0·62, 0·84	0·31	0·24, 0·40	0·21	0·15, 0·29	1·25	1·10, 1·41
Rural	85 062	0·11	0·09, 0·15	1·16	1·07, 1·26	17·5	17·2, 17·9	104 518	1·06	0·99, 1·14	0·53	0·48, 0·58	0·30	0·26, 0·34	1·89	1·79, 2·00
Social group																
Others	25 466	0·12	0·09, 0·18	1·00	0·86, 1·17	15·6	15·0, 16·3	29 344	0·75	0·64, 0·88	0·37	0·28, 0·49	0·25	0·18, 0·35	1·37	1·20, 1·56
Scheduled castes	22 978	0·11	0·06, 0·20	1·33	1·15, 1·55	18·1	17·5, 18·8	27 657	1·16	1·02, 1·32	0·53	0·44, 0·63	0·32	0·25, 0·41	2·00	1·82, 2·20
Scheduled tribes	22 384	0·10	0·05, 0·22	1·11	0·88, 1·40	19·1	18·2, 20·1	27 741	0·87	0·72, 1·05	0·56	0·44, 0·73	0·29	0·21, 0·41	1·72	1·50, 1·98
Other Backward Classes	49 546	0·10	0·07, 0·15	1·13	1·02, 1·26	16·7	16·3, 17·1	58 933	0·98	0·89, 1·08	0·45	0·39, 0·51	0·26	0·21, 0·31	1·69	1·57, 1·81
Religion																
Hinduism	92 966	0·11	0·08, 0·14	1·12	1·03, 1·21	17·2	16·9, 17·6	108 753	0·94	0·87, 1·01	0·47	0·42, 0·52	0·28	0·24, 0·32	1·68	1·59, 1·78
Islam	13 121	0·13	0·08, 0·22	1·32	1·09, 1·60	16·2	15·3, 17·1	17 856	1·19	1·00, 1·42	0·45	0·35, 0·59	0·29	0·22, 0·39	1·93	1·70, 2·20
Christianity	8554	0·04	0·01, 0·17	1·14	0·58, 2·23	15·4	13·4, 17·5	10 634	0·59	0·35, 0·98	0·18	0·09, 0·34	0·13	0·05, 0·34	0·89	0·61, 1·32
Others	5733	0·05	0·01, 0·20	1·13	0·71, 1·80	15·9	14·4, 17·5	6432	0·74	0·52, 1·05	0·46	0·26, 0·80	0·20	0·11, 0·38	1·40	1·06, 1·84
Economic group																
Poorest	20 303	0·17	0·09, 0·30	1·43	1·22, 1·67	19·5	18·8, 20·2	28 847	1·41	1·25, 1·60	0·67	0·56, 0·80	0·45	0·36, 0·55	2·53	2·32, 2·77
Poorer	25 054	0·11	0·07, 0·16	1·36	1·18, 1·55	18·1	17·4, 18·8	31 813	1·18	1·05, 1·34	0·59	0·50, 0·70	0·26	0·20, 0·34	2·04	1·86, 2·24
Middle	26 217	0·12	0·08, 0·18	1·07	0·92, 1·25	17·1	16·5, 17·8	30 458	0·95	0·82, 1·09	0·48	0·38, 0·60	0·31	0·23, 0·42	1·73	1·54, 1·94
Richer	25 116	0·07	0·04, 0·11	1·04	0·88, 1·22	17·3	16·6, 18·0	27 643	0·73	0·61, 0·87	0·27	0·21, 0·36	0·17	0·12, 0·26	1·18	1·03, 1·35
Richest	23 684	0·10	0·05, 0·20	0·92	0·76, 1·13	13·7	13·1, 14·4	24 914	0·52	0·41, 0·64	0·29	0·21, 0·39	0·17	0·13, 0·24	0·98	0·83, 1·14
State of residence																
Non-high focus	54 451	0·09	0·06, 0·14	0·99	0·88, 1·12	16·5	16·0, 16·9	60 010	0·63	0·55, 0·72	0·32	0·26, 0·39	0·16	0·12, 0·22	1·11	1·00, 1·23
High Focus	65 923	0·13	0·10, 0·17	1·33	1·23, 1·44	17·7	17·4, 18·1	83 665	1·29	1·20, 1·38	0·61	0·55, 0·67	0·38	0·33, 0·44	2·27	2·16, 2·40
Number of ANC visit																
≥ 4	74 404	0·09	0·07, 0·13	1·03	0·93, 1·14	16·4	16·0, 16·8	81 457	0·78	0·71, 0·87	0·37	0·32, 0·43	0·22	0·18, 0·26	1·37	1·27, 1·48
< 4	45 970	0·14	0·10, 0·21	1·36	1·23, 1·51	18·3	17·8, 18·8	62 218	1·23	1·13, 1·34	0·60	0·53, 0·68	0·36	0·30, 0·42	2·19	2·05, 2·33
Received supplementary food from *Anganwadi* centre																
Yes	74 804	0·09	0·07, 0·13	0·99	0·90, 1·09	17·0	16·6, 17·4	86 414	0·87	0·80, 0·95	0·44	0·39, 0·50	0·24	0·20, 0·29	1·55	1·45, 1·66
No	45 570	0·13	0·09, 0·18	1·38	1·23, 1·53	17·0	16·5, 17·6	57 261	1·08	0·98, 1·19	0·49	0·42, 0·57	0·32	0·27, 0·39	1·89	1·76, 2·04
Blood sample taken during ANC visit																
Yes	108 561	0·11	0·08, 0·14	1·12	1·04, 1·21	16·9	16·6, 17·2	122 716	0·90	0·83, 0·96	0·43	0·39, 0·48	0·25	0·21, 0·29	1·58	1·49, 1·67
No	11 813	0·13	0·07, 0·23	1·37	1·15, 1·63	18·4	17·6, 19·3	20 959	1·37	1·19, 1·56	0·64	0·52, 0·78	0·43	0·34, 0·56	2·44	2·21, 2·70
Institutional delivery																
Yes	111 987	0·09	0·07, 0·12	1·11	1·03, 1·20	16·9	16·6, 17·2	119 711	0·89	0·83, 0·96	0·44	0·40, 0·49	0·24	0·21, 0·27	1·57	1·49, 1·66
No	8387	0·32	0·15, 0·65	1·65	1·28, 2·12	19·3	18·1, 20·6	23 964	1·36	1·17, 1·57	0·57	0·46, 0·70	0·47	0·34, 0·65	2·40	2·14, 2·69
BMI of mother																
Optimum	57 779	0·09	0·06, 0·12	1·10	0·99, 1·22	16·6	16·1, 17·0	70 140	0·99	0·90, 1·09	0·52	0·46, 0·59	0·25	0·21, 0·30	1·76	1·64, 1·89
Underweight	26 835	0·16	0·10, 0·24	1·36	1·20, 1·54	20·8	20·2, 21·5	33 036	0·89	0·78, 1·02	0·49	0·40, 0·60	0·29	0·22, 0·37	1·67	1·51, 1·85
Overweight and obesity	35 760	0·11	0·06, 0·18	1·06	0·91, 1·22	14·9	14·3, 15·4	40 499	0·95	0·84, 1·08	0·34	0·27, 0·42	0·30	0·23, 0·39	1·59	1·44, 1·75
Sources of birth weight data																
From written card	65 147	0·06	0·04, 0·10	0·88	0·78, 0·98	16·1	15·7, 16·5	na	na		na		na		na	
From mother’s recall	55 227	0·17	0·13, 0·23	1·50	1·37, 1·65	18·2	17·8, 18·7	na	na		na		na		na	
Overall	120 374	0·11	0·09, 0·14	1·14	1·06, 1·23	17·0	16·7, 17·3	143 675	0·96	0·90, 1·02	0·46	0·42, 0·51	0·27	0·24, 0·31	1·69	1·61, 1·78

ANC, antenatal care; *P*, level of significance.

All *n* are unweighted.

Table 2 represents the association between maternal anaemia, IFA intake and ELBW, VLBW and LBW, with OR and 95 % CI estimated from the logistic regression model. Results showed a protective association for ELBW if a mother had consumed ≥ 100 IFA (OR: 0·54, CI: 0·31, 0·95, *P* = 0·032), as compared with a mother who did not buy/receive any IFA. A similar observation was noted in the case of VLBW (OR: 0·71, CI: 0·59, 0·84, *P* < 0·001) and LBW (OR: 0·84, CI: 0·80, 0·89, *P* < 0·001). Detailed results on various stages of LBW and its sub-categories with primary variables adjusted for confounders are available in the online supplement (see online supplementary material, Supplemental Table S3).

**Table 2:** Association between maternal iron-and-folic-acid (IFA) consumption and extremely low birth weight, very low birthweight and low birthweight

	Extremely low-birth weight*			Very low-birth weight*			Low-birth weight*		
	OR	95 % CI	*P*	OR	95 % CI	*P*	OR	95 % CI	*P*
No IFA	1·00 (referent)			1·00 (referent)			1·00 (referent)		
< 100 IFA	0·84	0·53, 1·34	0·473	0·94	0·81, 1·09	0·430	0·97	0·93, 1·02	0·194
≥ 100 IFA	0·54	0·31, 0·95	0·032	0·71	0·59, 0·84	< 0·001	0·84	0·80, 0·89	< 0·001

ANC, antenatal care, *P*, level of significance.

* Model is adjusted for IFA consumption, current age group of mother, mother’s age at first birth, education of mother, birth order, place of residence, social group, religion, economic group, state of residence, number of ANC visit, received supplementary food from *Anganwadi* centre, blood sample taken during ANC visit, institutional delivery, BMI of mother and sources of birth weight data.

The association between IFA consumption and neonatal mortality with timing of neonatal death is presented in Table 3. For neonatal mortality during day 0–1, the association with consumption of ≥ 100 IFA was protective (OR: 0·74, CI: 0·63, 0·88, *P* < 0·001), as compared with women who did not buy/receive any IFA. In case of neonatal death during day 2–6, no association with IFA consumption (*P* > 0·05) was observed. In the case of neonatal death during day 7–27 and death during day 0–27, multiple regression models showed a protective association for both groups of women – women who consumed < 100 IFA intake and women who consumed ≥ 100 IFA. A detailed analysis on the association between neonatal mortality and IFA consumption with adjusted confounders is presented in the online supplement (see online supplementary material, Supplemental Table S4).

**Table 3:** Association between iron-and-folic-acid (IFA) consumption and timing of neonatal mortality (day 0–1, day 2–6 and day 7–27) and neonatal mortality (day 0–27)

	Neonatal mortality (day 0–1)*			Neonatal mortality (day 2–6)*			Neonatal mortality (day 7–27)*			Neonatal mortality (day 0–27)*		
	OR	95 % CI	*P*	OR	95 % CI	*P*	OR	95 % CI	*P*	OR	95 % CI	*P*
No IFA	1·00 (referent)			1·00 (referent)			1·00 (referent)			1·00 (referent)		
< 100 IFA	0·89	0·78, 1·01	0·079	0·92	0·76, 1·12	0·431	0·74	0·59, 0·94	0·015	0·87	0·79, 0·96	0·006
≥ 100 IFA	0·74	0·63, 0·88	< 0·001	0·85	0·67, 1·07	0·171	0·62	0·46, 0·84	0·002	0·75	0·66, 0·84	< 0·001

ANC, antenatal care, *P*, level of significance.

* Model is adjusted for IFA consumption, current age group of mother, mother’s age at first birth, education of mother, sex of child, birth order, place of residence, social group, religion, economic group, state of residence, number of ANC visit, received supplementary food from *Anganwadi* centre, blood sample taken during ANC visit, institutional delivery and BMI of mother.

## Discussion

The current study aimed to understand the coverage of IFA consumption among prospective mothers and to assess the association between IFA consumption and various stages of low-birth weight and neonatal mortality in India. Aside from the protective association between ≥ 100 IFA consumption and LBW and neonatal mortality, the suboptimal increase in coverage (between 2005–2006 and 2015–2016) and a large variation in coverage of ≥ 100 IFA intake across 640 districts in India remain challenges to India’s public health system. Findings revealed that ≥ 100 IFA consumption by pregnant mothers was associated with reduced odds of ELBW, VLBW, LBW and neonatal death during day 0–1, day 7–27 and day 0–27. In addition, a protective association from neonatal death during day 7–27, and day 0–27 was observed for women who consumed < 100 IFA. No association between IFA consumption and neonatal death during day 2–6 was observed.

In the case of birth weight, multiple regression adjusted for potential confounders indicates that prevention of ELBW, VLBW and LBW were associated with IFA consumption by prospective mothers who consumed ≥ 100 IFA, but no association was observed for women who consumed < 100 IFA. The primary causes of ELBW and VLBW are PTB and intrauterine growth restriction, and the prevention of PTB and intrauterine growth restriction is multifactorial since biological pathways and preventive measures for these two conditions are different^(42)^. As to the role of IFA as a preventive measure against LBW, this finding is consistent with previous studies on India^(22,23)^ conducted using NFHS data, although these studies did not analyse various stages of LBW. Multiple regression analysis adjusted for potential confounders also indicates that women with no history of buying or receiving IFA had a higher likelihood of neonatal mortality and death at age day 0–1, day 7–27 and day 0–27 (neonatal mortality), as compared with women who consumed ≥ 100 IFA during their pregnancy. Existing population-based studies^(24,43,44)^ have concluded that lack of IFA consumption leads to neonatal mortality. A child’s death on day 0–1 among anaemic women indicates the importance of IFA as most neonatal deaths occur during the first three days of life^(28)^.

The current study acknowledges certain limitations that should be considered while interpreting the findings. First, data on all possible determinants (and unobservable determinants) of birth weight and neonatal mortality are not available with NFHS-4; thus, they could not be included in the analysis. For example, availability of information on PTB and intrauterine growth restriction would have been helpful in better correlating IFA consumption with LBW and neonatal mortality. Second, most information is self-reported, which might be affected by recall errors and social desirability bias. Third, IFA consists of Fe as well as folic acid, but as it is given as a combined fixed dose, the association may not be attributed to Fe alone^(22)^. Also, no details on the method of IFA consumption (e.g. timing of IFA intake) were captured in NFHS as this information would have been helpful in interpreting the association. Fourth, information on birth weight is based on data from health cards and mother’s recall, which reduced the sample size. However, the prevalence on LBW in the current study is comparable to the general population that offers confidence about the generalisability of the study findings. Fifth, supported by the sensitivity analysis, the multiple regression models adjusted for recording of birth weight data indicate that mothers’ recall had higher likelihood to record ELBW, VLBW and LBW (see online supplementary material, Supplemental Table S3). Sixth, a major limitation of the current study is the absence of data on anaemia status during pregnancy as this would enable an investigation into whether there is a modifying effect of anaemia on IFA–child health relationship, that is, if the association is stronger in anaemic women. Seventh and probably the most important limitation is that pregnant women have approximately twice the Fe demands during pregnancies, compared to non-pregnant women^(1)^. However without screening the need for Fe, if IFA is given to non-anaemic pregnant women, an overdose may lead to adverse pregnancy outcomes^(45)^. Also, according to the WHO, around 50 % of women aged 15–49 years are amenable to Fe supplementation to mitigate IDA^(46)^, and the other half of women might have anaemia from other causes (such as malaria, haemoglobinopathies, fluorosis and others) which cannot be treated with IFA supplementation. Finally, the current study used cross-sectional data and analysed the association between IFA and birth weight and neonatal mortality, thus the reader should refrain from drawing any causal inference from the study. Despite these limitations, the current study is the first of its kind to demonstrate the association between IFA consumption and various types of LBW and neonatal mortality using a nationally representative data set in India. Future study on this issue should investigate the effect of IFA supplementation separately on various types of LBW and neonatal mortality, where a comprehensive set of additional information on various socioeconomic and clinical parameters of pregnant mothers (e.g. Hb during pregnancy) are desired for discerning the effect of Fe and folic acid on LBW and neonatal deaths.

Based on the findings of the current study, it is encouraging to note the potential role of ≥ 100 IFA intake by prospective mothers in controlling various stages of LBW and neonatal mortality in India. However, the poor coverage of ≥ 100 IFA intake poses a threat to the success of the National Nutrition Mission. The government of India should reinforce the guidelines for distribution and consumption of IFA outlined for achieving the goals of the National Nutrition Mission.

## Supplementary material

For supplementary material accompanying this paper visit https://doi.org/10.1017/S1368980021004572. click here to view supplementary material
