# Supplementary material for: Maternal iron-and-folic-acid supplementation and its association with low-birth weight and neonatal mortality in India
Source: Public Health Nutr. 2021 Nov 8;25(3):623–33. doi: 10.1017/S1368980021004572 (PMC9991695; doi:10.1017/S1368980021004572)
Supplement: Supplementary file 1 [file S1368980021004572sup001.docx]

**Maternal Iron-and-Folic-Acid Supplementation and its Association with Low-birthweight and Neonatal Mortality in India**

**Online Supplementary material**

| **Content** | **Page no.** |
| --- | --- |
| **Table S1**: Change in prevalence (%) of ≥100 iron-and-folic-acid receipt between 2005-2006 and 2015-2016, in 29 states / union territories of India…………….…………………. | 3 |
| **Table S2**: Prevalence (%) of ≥100 iron-and-folic-acid receipt in 640 districts of 37 states / union territories of India, NFHS 2015-2016……………………………...……………. | 4 |
| **Table S3**: Association between iron-and-folic-acid (IFA) consumption and extremely low-birthweight, very low-birthweight, and low-birthweight……………………….............. | 21 |
| **Table S4**: Association between iron-and-folic-acid (IFA) consumption and timing of neonatal mortality (day 0-1, day 2-6, and day 7-27) and neonatal mortality (day 0-27) ………………………………………………………………………………………… | 23 |
| **Figure S1:** Directed Acyclic Graph (DAG) showing the causal pathways between exposure (IFA or iron-and-folic-acid tablet/ syrup intake during pregnancy) and outcome (birthweight) variable……………………………………………………………………… | 25 |
|  |  |

| **Table S1**. Change in prevalence (%) of ≥100 iron-and-folic-acid receipt, between 2005-2006 and 2015-2016, in 29 states / union territories of India. | | | | |
| --- | --- | --- | --- | --- |
|  | **NFHS 2005-2006** | **NFHS 2015-2016** | **Absolute change*** | **Relative change**** |
| Nagaland | 1.2 | 4.5 | 3.3 | 3.8 |
| Uttar Pradesh | 6.1 | 13.0 | 6.9 | 2.1 |
| Bihar | 6.4 | 9.8 | 3.4 | 1.5 |
| Meghalaya | 6.4 | 38.0 | 31.6 | 5.9 |
| Manipur | 7.0 | 40.1 | 33.1 | 5.7 |
| Madhya Pradesh | 7.2 | 23.9 | 16.7 | 3.3 |
| Arunachal Pradesh | 7.6 | 9.0 | 1.4 | 1.2 |
| Rajasthan | 8.8 | 17.4 | 8.6 | 2.0 |
| Jharkhand | 9.7 | 15.5 | 5.8 | 1.6 |
| Chhattisgarh | 10.5 | 30.6 | 20.1 | 2.9 |
| Assam | 10.8 | 32.8 | 22.0 | 3.0 |
| Tripura | 12.0 | 13.6 | 1.6 | 1.1 |
| Punjab | 13.8 | 42.9 | 29.1 | 3.1 |
| West Bengal | 14.8 | 28.2 | 13.4 | 1.9 |
| Uttarakhand | 16.9 | 25.2 | 8.3 | 1.5 |
| Jammu & Kashmir (including Ladakh)^1^ | 17.4 | 31.0 | 13.6 | 1.8 |
| Haryana | 18.2 | 32.7 | 14.5 | 1.8 |
| Mizoram | 18.6 | 54.8 | 36.2 | 2.9 |
| Maharashtra | 19.6 | 40.9 | 21.3 | 2.1 |
| Odisha | 23.5 | 37.4 | 13.9 | 1.6 |
| Gujarat | 25.6 | 37.7 | 12.1 | 1.5 |
| Sikkim | 26.6 | 53.1 | 26.5 | 2.0 |
| Andhra Pradesh (including Telangana)^2^ | 27.1 | 55.7 | 28.6 | 2.1 |
| Himachal Pradesh | 27.2 | 50.2 | 23.0 | 1.8 |
| Karnataka | 28.9 | 46.4 | 17.5 | 1.6 |
| Tamil Nadu | 28.9 | 64.7 | 35.8 | 2.2 |
| Delhi | 30.5 | 55.1 | 24.6 | 1.8 |
| Goa | 63.7 | 67.8 | 4.1 | 1.1 |
| Kerala | 81.7 | 74.8 | -6.9 | 0.9 |
|  |  |  |  |  |
| India | 15.6 | 30.7 | 15.1 | 2.0 |
| * Absolute change is the difference (NFHS 2015-2016 *minus* NFHS 2005-2006) of prevalence.  ** Relative change is the ratio (NFHS 2015-2016 / NFHS 2005-2006) of prevalence.  ^1^During survey period of the 2005–2006 National Family Health Survey, Ladakh was part of Jammu & Kashmir.  ^2^During survey period of the 2005–2006 National Family Health Survey, Telangana was part of Andhra Pradesh.  NFHS: National Family Health Survey. | | | | |

| **Table S2.** Prevalence (%) of ≥100 iron-and-folic-acid receipt in 640 districts of 37 states / union territories of India, NFHS 2015-2016. | |
| --- | --- |
| **Andaman & Nicobar Islands** |  |
| Nicobars | 20.3 |
| North & Middle Andaman | 45.7 |
| South Andaman | 68.0 |
|  |  |
| **Andhra Pradesh** |  |
| Guntur | 39.2 |
| West Godavari | 44.8 |
| Prakasam | 49.0 |
| Kurnool | 54.6 |
| Vizianagaram | 55.9 |
| Visakhapatnam | 57.1 |
| Kadapa | 57.8 |
| Anantapur | 59.6 |
| East Godavari | 61.1 |
| Chittoor | 64.1 |
| Srikakulam | 65.7 |
| Nellore | 69.1 |
| Krishna | 71.3 |
|  |  |
| **Arunachal Pradesh** |  |
| West Siang | 1.6 |
| Upper Subansiri | 1.8 |
| East Kameng | 2.0 |
| Kurung Kumey | 2.9 |
| Anjaw | 4.8 |
| East Siang | 7.0 |
| West Kameng | 8.0 |
| Tawang | 9.3 |
| Tirap | 9.7 |
| Upper Siang | 9.9 |
| Changlang | 11.2 |
| Lower Subansiri | 11.4 |
| Lower Dibang Valley | 11.8 |
| Papumpare | 12.5 |
| Lohit | 14.4 |
| Dibang Valley | 25.8 |
|  |  |
| **Assam** |  |
| Dhubri | 13.8 |
| Karimganj | 18.3 |
| Cachar | 19.2 |
| Barpeta | 19.3 |
| Kamrup | 24.2 |
| Hailakandi | 24.3 |
| Darrang | 25.1 |
| Karbi Anglong | 27.0 |
| Kokrajhar | 27.6 |
| Udalguri | 30.5 |
| Goalpara | 31.9 |
| Nagaon | 32.1 |
| Nalbari | 33.9 |
| Chirang | 34.6 |
| Dima Hasao | 34.8 |
| Dhemaji | 35.3 |
| Bongaigaon | 36.1 |
| Morigaon | 38.2 |
| Baksa | 40.1 |
| Tinsukia | 40.3 |
| Sonitpur | 40.7 |
| Sivasagar | 42.9 |
| Golaghat | 45.2 |
| Lakhimpur | 46.3 |
| Kamrup Metropolitan | 47.2 |
| Dibrugarh | 57.4 |
| Jorhat | 66.3 |
|  |  |
| **Bihar** |  |
| Sheohar | 2.6 |
| Madhepura | 2.6 |
| Kaimur (Bhabua) | 3.4 |
| Purba Champaran | 3.9 |
| Lakhisarai | 4.5 |
| Sitamarhi | 4.7 |
| Muzaffarpur | 4.8 |
| Gaya | 5.7 |
| Begusarai | 5.9 |
| Khagaria | 6.0 |
| Arwal | 6.0 |
| Katihar | 6.7 |
| Darbhanga | 6.8 |
| Rohtas | 7.2 |
| Nalanda | 7.3 |
| Saran | 7.5 |
| Aurangabad | 7.8 |
| Araria | 8.0 |
| Supaul | 8.2 |
| Nawada | 8.8 |
| Buxar | 9.3 |
| Purnia | 9.6 |
| Samastipur | 9.8 |
| Munger | 10.2 |
| Bhojpur | 10.4 |
| Sheikhpura | 10.5 |
| Saharsa | 12.5 |
| Jamui | 12.7 |
| Jehanabad | 13.4 |
| Madhubani | 13.5 |
| Gopalganj | 14.2 |
| Pashchim Champaran | 14.4 |
| Vaishali | 14.7 |
| Kishanganj | 15.4 |
| Banka | 17.1 |
| Bhagalpur | 17.6 |
| Siwan | 20.0 |
| Patna | 21.2 |
|  |  |
| **Chandigarh** | 44.9 |
|  |  |
| **Chhattisgarh** |  |
| Surguja | 18.5 |
| Janjgir - Champa | 18.6 |
| Kabirdham | 19.0 |
| Raigarh | 21.3 |
| Bijapur | 21.3 |
| Narayanpur | 23.7 |
| Mahasamund | 23.8 |
| Korba | 24.3 |
| Jashpur | 26.1 |
| Dantewada | 26.2 |
| Bastar | 29.3 |
| Raipur | 31.7 |
| Durg | 37.6 |
| Kanker | 38.5 |
| Bilaspur | 39.4 |
| Koriya | 39.6 |
| Rajnandgaon | 40.0 |
| Dhamtari | 42.9 |
|  |  |
|  |  |
| **Dadra & Nagar Haveli** | 44.3 |
|  |  |
| **Daman & Diu** |  |
| Daman | 44.0 |
| Diu | 50.3 |
|  |  |
| **Delhi** |  |
| North East | 42.0 |
| North | 42.4 |
| East | 43.3 |
| West | 52.1 |
| South | 52.3 |
| North West | 53.9 |
| New Delhi | 58.6 |
| Central | 70.2 |
| South West | 72.4 |
|  |  |
| **Goa** |  |
| North Goa | 88.6 |
| South Goa | 35.8 |
|  |  |
| **Gujarat** |  |
| Dahod | 14.2 |
| Panchmahal | 20.8 |
| Banaskantha | 23.5 |
| Patan | 24.4 |
| Surendranagar | 28.0 |
| Sabarkantha | 28.1 |
| Junagadh | 28.3 |
| Amreli | 32.1 |
| Bhavnagar | 32.4 |
| Kheda | 32.9 |
| Mahesana | 34.5 |
| Surat | 37.7 |
| Vadodara | 38.1 |
| Kachchh | 38.4 |
| Dang | 39.7 |
| Anand | 40.3 |
| Valsad | 41.1 |
| Rajkot | 42.2 |
| Tapi | 43.5 |
| Porbandar | 43.9 |
| Jamnagar | 44.8 |
| Narmada | 45.6 |
| Gandhinagar | 45.8 |
| Bharuch | 52.0 |
| Ahmadabad | 57.3 |
| Navsari | 59.3 |
|  |  |
| **Haryana** |  |
| Mewat | 6.4 |
| Palwal | 8.2 |
| Faridabad | 19.2 |
| Gurgaon | 19.4 |
| Rewari | 24.5 |
| Rohtak | 28.0 |
| Sonipat | 29.9 |
| Jhajjar | 30.5 |
| Panipat | 31.8 |
| Bhiwani | 32.0 |
| Hisar | 32.9 |
| Jind | 33.2 |
| Mahendragarh | 35.6 |
| Kurukshetra | 39.6 |
| Sirsa | 41.2 |
| Ambala | 48.2 |
| Fatehabad | 49.3 |
| Panchkula | 51.9 |
| Kaithal | 54.9 |
| Karnal | 57.8 |
| Yamunanagar | 61.5 |
|  |  |
| **Himachal Pradesh** |  |
| Mandi | 34.5 |
| Chamba | 39.3 |
| Lahul and Spiti | 46.0 |
| Sirmaur | 47.7 |
| Solan | 49.0 |
| Kinnaur | 50.4 |
| Una | 53.6 |
| Hamirpur | 53.8 |
| Shimla | 55.3 |
| Bilaspur | 57.0 |
| Kullu | 57.2 |
| Kangra | 59.6 |
|  |  |
| **Jammu & Kashmir** |  |
| Doda | 9.2 |
| Anantnag | 11.9 |
| Ganderbal | 13.1 |
| Bandipore | 16.2 |
| Kishtwar | 17.3 |
| Ramban | 18.1 |
| Kupwara | 20.2 |
| Badgam | 20.2 |
| Kulgam | 20.6 |
| Rajouri | 21.4 |
| Shupiyan | 21.8 |
| Srinagar | 27.6 |
| Pulwama | 30.4 |
| Baramula | 31.9 |
| Reasi | 37.0 |
| Punch | 41.1 |
| Samba | 49.5 |
| Udhampur | 50.7 |
| Kathua | 57.1 |
| Jammu | 57.5 |
|  |  |
| **Jharkhand** |  |
| Garhwa | 3.4 |
| Chatra | 6.9 |
| Simdega | 7.3 |
| Sahibganj | 7.5 |
| Latehar | 8.5 |
| Godda | 8.8 |
| Pakur | 9.2 |
| Palamu | 9.3 |
| Giridih | 9.7 |
| Pashchimi Singhbhum | 10.9 |
| Kodarma | 11.1 |
| Hazaribagh | 13.5 |
| Dumka | 14.3 |
| Jamtara | 14.4 |
| Purbi Singhbhum | 15.9 |
| Ramgarh | 19.5 |
| Gumla | 20.0 |
| Dhanbad | 20.7 |
| Deoghar | 20.8 |
| Lohardaga | 23.2 |
| Bokaro | 24.2 |
| Saraikela Kharsawan | 24.8 |
| Khunti | 24.9 |
| Ranchi | 35.1 |
|  |  |
| **Karnataka** |  |
| Mandya | 19.9 |
| Chikmagalur | 23.2 |
| Koppal | 26.1 |
| Yadgir | 26.5 |
| Haveri | 28.3 |
| Gulbarga | 34.3 |
| Ramanagara | 36.2 |
| Uttara kannada | 38.1 |
| Kodagu | 38.4 |
| Bijapur | 39.1 |
| Hassan | 39.7 |
| Shimoga | 40.4 |
| Udupi | 40.8 |
| Dakshina Kannada | 41.9 |
| Gadag | 44.1 |
| Bellary | 45.3 |
| Chitradurga | 45.8 |
| Dharwad | 46.4 |
| Bagalkot | 46.6 |
| Bangalore Rural | 46.7 |
| Chikkaballapura | 48.0 |
| Bidar | 49.4 |
| Bangalore | 50.9 |
| Mysore | 51.9 |
| Chamarajanagar | 53.7 |
| Raichur | 54.6 |
| Tumkur | 55.1 |
| Kolar | 61.5 |
| Belgaum | 62.0 |
| Davanagere | 66.6 |
|  |  |
| **Kerala** |  |
| Kottayam | 61.0 |
| Thiruvananthapuram | 61.0 |
| Alappuzha | 65.8 |
| Kollam | 66.2 |
| Malappuram | 71.3 |
| Idukki | 72.2 |
| Ernakulam | 72.9 |
| Pathanamthitta | 74.0 |
| Wayanad | 77.2 |
| Thrissur | 80.6 |
| Kannur | 85.8 |
| Kasaragod | 86.8 |
| Kozhikode | 86.9 |
| Palakkad | 88.4 |
|  |  |
| **Lakshadweep** | 85.5 |
|  |  |
| **Ladakh** |  |
| Kargil | 29.3 |
| Leh | 31.7 |
|  |  |
| **Madhya Pradesh** |  |
| Sidhi | 10.4 |
| Alirajpur | 12.7 |
| Tikamgarh | 14.2 |
| Rewa | 14.2 |
| Dhar | 14.2 |
| Mandsaur | 15.0 |
| Vidisha | 15.6 |
| Panna | 16.3 |
| Datia | 16.6 |
| Shivpuri | 16.6 |
| Umaria | 16.6 |
| Chhatarpur | 16.9 |
| Rajgarh | 17.3 |
| Satna | 17.5 |
| Sagar | 18.0 |
| Morena | 18.2 |
| Ashoknagar | 18.2 |
| Singrauli | 19.1 |
| Ujjain | 19.3 |
| Dindori | 19.3 |
| Jhabua | 19.7 |
| Barwani | 20.1 |
| Sehore | 20.5 |
| Shahdol | 21.1 |
| Guna | 21.3 |
| Damoh | 21.6 |
| Sheopur | 21.7 |
| Harda | 21.7 |
| Bhind | 23.3 |
| Raisen | 23.3 |
| Neemuch | 23.6 |
| Ratlam | 23.6 |
| Khargone (West Nimar) | 24.1 |
| Dewas | 25.7 |
| Hoshangabad | 26.8 |
| Betul | 27.8 |
| Mandla | 27.9 |
| Burhanpur | 28.9 |
| Shajapur | 29.3 |
| Katni | 29.8 |
| Anuppur | 30.9 |
| Gwalior | 33.6 |
| Balaghat | 33.7 |
| Narsimhapur | 34.1 |
| Khandwa (East Nimar) | 34.6 |
| Indore | 35.4 |
| Seoni | 37.4 |
| Chhindwara | 38.2 |
| Bhopal | 39.0 |
| Jabalpur | 44.0 |
|  |  |
| **Maharashtra** |  |
| Dhule | 15.8 |
| Aurangabad | 19.8 |
| Buldana | 21.6 |
| Ahmadnagar | 23.5 |
| Bid | 23.6 |
| Sindhudurg | 29.4 |
| Parbhani | 31.1 |
| Satara | 33.7 |
| Akola | 33.8 |
| Yavatmal | 35.7 |
| Mumbai Suburban | 37.1 |
| Osmanabad | 37.6 |
| Washim | 37.7 |
| Nanded | 39.3 |
| Sangli | 39.6 |
| Nandurbar | 39.7 |
| Jalgaon | 39.7 |
| Bhandara | 40.7 |
| Latur | 40.7 |
| Nashik | 41.2 |
| Hingoli | 42.8 |
| Thane | 44.5 |
| Wardha | 45.2 |
| Jalna | 46.0 |
| Kolhapur | 46.7 |
| Chandrapur | 47.0 |
| Raigarh | 47.3 |
| Gadchiroli | 48.9 |
| Amravati | 50.2 |
| Solapur | 50.9 |
| Nagpur | 52.7 |
| Mumbai | 52.7 |
| Ratnagiri | 53.5 |
| Pune | 55.9 |
| Gondiya | 71.7 |
|  |  |
| **Manipur** |  |
| Senapati | 14.2 |
| Ukhrul | 14.8 |
| Tamenglong | 15.7 |
| Churachandpur | 22.0 |
| Chandel | 26.4 |
| Bishnupur | 45.6 |
| Imphal east | 46.3 |
| Thoubal | 49.0 |
| Imphal west | 54.9 |
|  |  |
| **Meghalaya** |  |
| West Garo Hills | 14.4 |
| East Garo Hills | 20.8 |
| West Khasi Hills | 33.0 |
| Ribhoi | 35.7 |
| Jaintia Hills | 44.5 |
| South Garo Hills | 49.0 |
| East Khasi Hills | 60.5 |
|  |  |
| **Mizoram** |  |
| Lunglei | 40.8 |
| Lawngtlai | 42.9 |
| Mamit | 52.0 |
| Champhai | 53.7 |
| Saiha | 54.8 |
| Kolasib | 59.3 |
| Aizawl | 62.0 |
| Serchhip | 62.9 |
|  |  |
| **Nagaland** |  |
| Zunheboto | 0.0 |
| Longleng | 0.4 |
| Mon | 0.8 |
| Phek | 0.9 |
| Kiphire | 1.4 |
| Tuensang | 2.4 |
| Mokokchung | 7.6 |
| Peren | 7.6 |
| Dimapur | 8.1 |
| Kohima | 8.7 |
| Wokha | 10.0 |
|  |  |
| **Odisha** |  |
| Baleshwar | 18.4 |
| Gajapati | 22.7 |
| Ganjam | 23.8 |
| Kendrapara | 24.6 |
| Bhadrak | 29.1 |
| Cuttack | 31.4 |
| Koraput | 32.3 |
| Kalahandi | 33.5 |
| Khordha | 34.5 |
| Debagarh | 35.2 |
| Mayurbhanj | 36.2 |
| Nabarangapur | 36.3 |
| Dhenkanal | 36.9 |
| Anugul | 38.4 |
| Nayagarh | 39.0 |
| Malkangiri | 39.9 |
| Baudh | 42.6 |
| Sundargarh | 42.8 |
| Bargarh | 43.5 |
| Nuapada | 43.7 |
| Sambalpur | 43.9 |
| Jajapur | 45.0 |
| Kandhamal | 45.8 |
| Kendujhar | 46.5 |
| Jagatsinghapur | 47.0 |
| Puri | 49.0 |
| Jharsuguda | 50.1 |
| Balangir | 50.1 |
| Subarnapur | 50.2 |
| Rayagada | 52.5 |
|  |  |
| **Puducherry** |  |
| Karaikal | 65.0 |
| Puducherry | 65.6 |
| Yanam | 78.1 |
| Mahe | 89.5 |
|  |  |
| **Punjab** |  |
| Firozpur | 26.0 |
| Mansa | 32.3 |
| Shahid Bhagat Singh Nagar | 33.9 |
| Hoshiarpur | 35.8 |
| Patiala | 37.4 |
| Bathinda | 37.8 |
| Barnala | 39.9 |
| Kapurthala | 40.1 |
| Fatehgarh Sahib | 40.3 |
| Faridkot | 40.7 |
| Moga | 41.0 |
| Ludhiana | 42.4 |
| Amritsar | 43.9 |
| Rupnagar | 47.3 |
| Muktsar | 48.4 |
| Sangrur | 48.5 |
| Gurdaspur | 52.4 |
| Jalandhar | 53.6 |
| Tarn Taran | 57.6 |
| Sahibzada Ajit Singh Nagar | 61.5 |
|  |  |
| **Rajasthan** |  |
| Bharatpur | 5.3 |
| Sawai Madhopur | 8.2 |
| Rajsamand | 8.6 |
| Baran | 8.6 |
| Dungarpur | 8.9 |
| Ajmer | 9.1 |
| Bikaner | 10.5 |
| Jaisalmer | 10.5 |
| Barmer | 10.7 |
| Bundi | 10.8 |
| Nagaur | 11.8 |
| Karauli | 12.2 |
| Dhaulpur | 12.9 |
| Alwar | 13.0 |
| Banswara | 13.9 |
| Jodhpur | 14.8 |
| Hanumangarh | 15.6 |
| Pali | 16.2 |
| Chittaurgarh | 16.2 |
| Sikar | 16.6 |
| Churu | 17.9 |
| Dausa | 18.1 |
| Sirohi | 18.7 |
| Jhalawar | 19.6 |
| Tonk | 19.9 |
| Udaipur | 19.9 |
| Jalor | 21.6 |
| Pratapgarh | 30.8 |
| Jhunjhunun | 30.9 |
| Jaipur | 31.4 |
| Kota | 31.6 |
| Bhilwara | 31.7 |
| Ganganagar | 31.8 |
|  |  |
| **Sikkim** |  |
| East District | 42.7 |
| South District | 63.3 |
| West District | 66.8 |
| North District | 70.4 |
|  |  |
| **Tamil Nadu** |  |
| Virudhunagar | 37.9 |
| Tirunelveli | 45.3 |
| Dharmapuri | 51.1 |
| Ariyalur | 52.3 |
| Thoothukkudi | 53.1 |
| Nagapattinam | 54.6 |
| Ramanathapuram | 54.9 |
| Theni | 56.6 |
| Nilgiris | 57.3 |
| Perambalur | 57.3 |
| Tiruvannamalai | 58.9 |
| Tiruchirappalli | 60.4 |
| Kancheepuram | 60.8 |
| Viluppuram | 62.2 |
| Pudukkottai | 63.1 |
| Thanjavur | 64.6 |
| Salem | 65.0 |
| Cuddalore | 65.5 |
| Namakkal | 66.8 |
| Madurai | 66.9 |
| Dindigul | 67.5 |
| Tiruppur | 68.1 |
| Erode | 68.3 |
| Vellore | 69.6 |
| Chennai | 70.2 |
| Sivaganga | 70.6 |
| Thiruvallur | 71.4 |
| Kanniyakumari | 71.9 |
| Thiruvarur | 72.5 |
| Karur | 73.4 |
| Coimbatore | 75.6 |
| Krishnagiri | 81.6 |
|  |  |
| **Tripura** |  |
| North Tripura | 12.6 |
| Dhalai | 13.2 |
| West Tripura | 13.9 |
| South Tripura | 14.2 |
|  |  |
| **Uttar Pradesh** |  |
| Kanshiram Nagar | 2.0 |
| Shrawasti | 2.6 |
| Auraiya | 3.1 |
| Rampur | 4.9 |
| Unnao | 5.1 |
| Sitapur | 5.2 |
| Bahraich | 5.7 |
| Gonda | 5.9 |
| Fatehpur | 6.2 |
| Moradabad | 6.4 |
| Balrampur | 6.4 |
| Jhansi | 6.5 |
| Basti | 6.5 |
| Sant Kabir Nagar | 6.5 |
| Mathura | 6.6 |
| Mau | 6.6 |
| Banda | 6.8 |
| Kanpur Dehat | 6.9 |
| Mainpuri | 7.2 |
| Firozabad | 7.7 |
| Etawah | 7.8 |
| Faizabad | 7.8 |
| Mahoba | 7.9 |
| Jyotiba Phule Nagar | 8.6 |
| Bulandshahr | 9.2 |
| Farrukhabad | 9.2 |
| Bareilly | 9.5 |
| Kaushambi | 9.7 |
| Ghazipur | 9.7 |
| Sultanpur | 9.9 |
| Ballia | 10.1 |
| Bara banki | 10.2 |
| Azamgarh | 10.6 |
| Kannauj | 10.7 |
| Etah | 10.7 |
| Jalaun | 10.9 |
| Siddharth nagar | 10.9 |
| Aligarh | 11.6 |
| Lalitpur | 11.8 |
| Shahjahanpur | 12.1 |
| Budaun | 12.4 |
| Hardoi | 13.2 |
| Mahamaya Nagar | 13.3 |
| Agra | 13.7 |
| Hamirpur | 14.0 |
| Bijnor | 14.2 |
| Kheri | 14.3 |
| Saharanpur | 14.7 |
| Ambedkar Nagar | 15.2 |
| Sant Ravidas Nagar | 15.5 |
| Pratapgarh | 16.4 |
| Deoria | 16.6 |
| Meerut | 16.8 |
| Muzaffarnagar | 17.0 |
| Jaunpur | 17.1 |
| Pilibhit | 17.3 |
| Chitrakoot | 17.4 |
| Ghaziabad | 17.6 |
| Gorakhpur | 17.8 |
| Lucknow | 19.7 |
| Varanasi | 20.3 |
| Gautam Buddha Nagar | 20.7 |
| Chandauli | 21.2 |
| Rae Bareli | 21.4 |
| Sonbhadra | 21.7 |
| Mahrajganj | 22.0 |
| Mirzapur | 22.5 |
| Kushinagar | 24.2 |
| Allahabad | 24.7 |
| Baghpat | 26.0 |
| Kanpur Nagar | 26.3 |
|  |  |
| **Uttarakhand** |  |
| Udham Singh Nagar | 16.8 |
| Chamoli | 19.0 |
| Hardwar | 19.3 |
| Bageshwar | 23.9 |
| Rudraprayag | 24.5 |
| Tehri Garhwal | 25.0 |
| Garhwal | 26.4 |
| Champawat | 27.7 |
| Uttarkashi | 28.1 |
| Pithoragarh | 28.7 |
| Dehradun | 29.6 |
| Almora | 35.7 |
| Nainital | 41.4 |
|  |  |
| **West Bengal** |  |
| Uttar Dinajpur | 6.1 |
| Maldah | 19.3 |
| Kochbihar | 20.5 |
| North Twenty-Four Parganas | 21.5 |
| Murshidabad | 22.7 |
| Birbhum | 22.7 |
| South Twenty-Four Parganas | 23.8 |
| Jalpaiguri | 24.2 |
| Paschim Medinipur | 26.2 |
| Purba Medinipur | 29.6 |
| Hugli | 33.4 |
| Puruliya | 34.9 |
| Nadia | 36.4 |
| Barddhaman | 36.8 |
| Haora | 38.6 |
| Dakshin Dinajpur | 39.6 |
| Kolkata | 42.1 |
| Darjiling | 42.6 |
| Bankura | 46.7 |
|  |  |
| **Telangana** |  |
| Nalgonda | 36.5 |
| Medak | 37.3 |
| Adilabad | 41.9 |
| Mahbubnagar | 49.1 |
| Karimnagar | 49.4 |
| Nizamabad | 49.6 |
| Rangareddy | 54.0 |
| Khammam | 57.0 |
| Warangal | 70.4 |
| Hyderabad | 72.3 |
|  |  |
| NFHS: National Family Health Survey. | |

| **Table S3**. Association between iron-and-folic-acid (IFA) consumption and extremely low-birthweight, very low-birthweight, and low-birthweight. | | | | | | |
| --- | --- | --- | --- | --- | --- | --- |
|  | **Extremely low-birthweight** | | **Very low-birthweight** | | **Low-birthweight** | |
|  | OR (95% CI) | p | OR (95% CI) | p | OR (95% CI) | p |
| **IFA consumption** |  |  |  |  |  |  |
| No IFA | **1.00 (referent)** |  | **1.00 (referent)** |  | **1.00 (referent)** |  |
| <100 IFA | **0.84 (0.53-1.34)** | **0.473** | **0.94 (0.81-1.09)** | **0.430** | **0.97 (0.93-1.02)** | **0.194** |
| ≥100 IFA | **0.54 (0.31-0.95)** | **0.032** | **0.71 (0.59-0.84)** | **<0.001** | **0.84 (0.80-0.89)** | **<0.001** |
| **Current age-group of mother** |  |  |  |  |  |  |
| 15-19 | 1.00 (referent) |  | 1.00 (referent) |  | 1.00 (referent) |  |
| 20-29 | 1.18 (0.41-3.41) | 0.766 | 0.67 (0.51-0.88) | 0.004 | 0.87 (0.80-0.95) | 0.001 |
| 30-39 | 1.20 (0.37-3.90) | 0.760 | 0.57 (0.41-0.78) | <0.001 | 0.85 (0.78-0.94) | 0.001 |
| ≥40 | 0.52 (0.05-5.17) | 0.575 | 0.68 (0.42-1.10) | 0.119 | 0.83 (0.72-0.97) | 0.017 |
| **Mother’s age at first birth** |  |  |  |  |  |  |
| <18 | 1.00 (referent) |  | 1.00 (referent) |  | 1.00 (referent) |  |
| ≥18 | 0.97 (0.53-1.79) | 0.934 | 1.13 (0.93-1.36) | 0.222 | 0.96 (0.91-1.02) | 0.169 |
| **Education of mother** |  |  |  |  |  |  |
| No or incomplete primary | 1.00 (referent) |  | 1.00 (referent) |  | 1.00 (referent) |  |
| Primary or incomplete secondary | 0.99 (0.63-1.56) | 0.974 | 0.86 (0.75-0.98) | 0.027 | 0.91 (0.88-0.95) | <0.001 |
| Secondary or higher | 0.66 (0.34-1.28) | 0.224 | 0.66 (0.54-0.80) | <0.001 | 0.74 (0.70-0.79) | <0.001 |
| **Birth order** |  |  |  |  |  |  |
| 1 | 1.00 (referent) |  | 1.00 (referent) |  | 1.00 (referent) |  |
| 2 | 0.85 (0.55-1.32) | 0.469 | 0.85 (0.74-0.97) | 0.017 | 0.86 (0.83-0.90) | <0.001 |
| 3 | 0.84 (0.47-1.50) | 0.560 | 0.87 (0.73-1.04) | 0.123 | 0.86 (0.82-0.90) | <0.001 |
| 4 | 0.95 (0.44-2.04) | 0.886 | 1.20 (0.95-1.51) | 0.126 | 0.89 (0.83-0.95) | 0.001 |
| ≥5 | 0.73 (0.27-1.97) | 0.534 | 0.93 (0.70-1.25) | 0.646 | 0.88 (0.81-0.96) | 0.003 |
| **Place of residence** |  |  |  |  |  |  |
| Urban | 1.00 (referent) |  | 1.00 (referent) |  | 1.00 (referent) |  |
| Rural | 0.86 (0.55-1.35) | 0.523 | 0.87 (0.76-1.00) | 0.058 | 0.93 (0.89-0.96) | <0.001 |
| **Social group** |  |  |  |  |  |  |
| Others | 1.00 (referent) |  | 1.00 (referent) |  | 1.00 (referent) |  |
| Scheduled castes | 0.71 (0.40-1.23) | 0.219 | 1.21 (1.02-1.44) | 0.031 | 1.11 (1.05-1.16) | <0.001 |
| Scheduled tribes | 0.46 (0.22-0.96) | 0.037 | 0.76 (0.61-0.94) | 0.012 | 0.94 (0.88-0.99) | 0.023 |
| Other Backward Classes | 0.68 (0.44-1.05) | 0.084 | 1.00 (0.86-1.16) | 0.969 | 0.99 (0.95-1.03) | 0.572 |
| **Religion** |  |  |  |  |  |  |
| Hinduism | 1.00 (referent) |  | 1.00 (referent) |  | 1.00 (referent) |  |
| Islam | 1.32 (0.80-2.17) | 0.272 | 1.07 (0.91-1.27) | 0.410 | 0.95 (0.90-1.00) | 0.035 |
| Christianity | 0.31 (0.07-1.41) | 0.130 | 0.36 (0.24-0.55) | <0.001 | 0.49 (0.45-0.54) | <0.001 |
| Others | 0.36 (0.09-1.50) | 0.162 | 1.08 (0.82-1.42) | 0.578 | 0.86 (0.79-0.93) | <0.001 |
| **Economic group** |  |  |  |  |  |  |
| Poorest | 1.00 (referent) |  | 1.00 (referent) |  | 1.00 (referent) |  |
| Poorer | 0.92 (0.51-1.64) | 0.771 | 1.08 (0.91-1.28) | 0.375 | 0.96 (0.92-1.01) | 0.163 |
| Middle | 1.08 (0.60-1.97) | 0.791 | 0.93 (0.77-1.12) | 0.438 | 0.96 (0.91-1.02) | 0.188 |
| Richer | 0.74 (0.37-1.49) | 0.398 | 0.96 (0.78-1.18) | 0.674 | 1.03 (0.97-1.09) | 0.340 |
| Richest | 0.91 (0.42-1.98) | 0.812 | 0.87 (0.68-1.10) | 0.244 | 0.92 (0.85-0.98) | 0.011 |
| **State of residence** |  |  |  |  |  |  |
| Non-high focus | 1.00 (referent) |  | 1.00 (referent) |  | 1.00 (referent) |  |
| High Focus | 0.87 (0.58-1.32) | 0.524 | 1.05 (0.93-1.20) | 0.424 | 1.00 (0.97-1.04) | 0.928 |
| **Number of ANC visit** |  |  |  |  |  |  |
| ≥4 | 1.00 (referent) |  | 1.00 (referent) |  | 1.00 (referent) |  |
| <4 | 1.08 (0.72-1.60) | 0.719 | 1.02 (0.90-1.15) | 0.807 | 1.03 (1.00-1.07) | 0.070 |
| **Received supplementary food from *Anganwadi* centre** |  |  |  |  |  |  |
| Yes | 1.00 (referent) |  | 1.00 (referent) |  | 1.00 (referent) |  |
| No | 1.27 (0.86-1.86) | 0.225 | 1.36 (1.21-1.53) | <0.001 | 1.03 (1.00-1.07) | 0.052 |
| **Blood sample taken during ANC visit** |  |  |  |  |  |  |
| Yes | 1.00 (referent) |  | 1.00 (referent) |  | 1.00 (referent) |  |
| No | 0.85 (0.47-1.55) | 0.598 | 0.93 (0.78-1.12) | 0.452 | 0.97 (0.92-1.02) | 0.223 |
| **Institutional delivery** |  |  |  |  |  |  |
| Yes | 1.00 (referent) |  | 1.00 (referent) |  | 1.00 (referent) |  |
| No | 1.74 (0.98-3.10) | 0.058 | 1.19 (0.97-1.46) | 0.093 | 1.11 (1.04-1.17) | 0.001 |
| **BMI of mother** |  |  |  |  |  |  |
| Optimum | 1.00 (referent) |  | 1.00 (referent) |  | 1.00 (referent) |  |
| Underweight | 1.71 (1.13-2.60) | 0.012 | 1.28 (1.12-1.46) | <0.001 | 1.29 (1.24-1.34) | <0.001 |
| Overweight and obesity | 1.09 (0.68-1.73) | 0.728 | 1.06 (0.92-1.21) | 0.434 | 0.92 (0.89-0.96) | <0.001 |
| **Sources of birthweight data** |  |  |  |  |  |  |
| From written card | 1.00 (referent) |  | 1.00 (referent) |  | 1.00 (referent) |  |
| From mother’s recall | 2.41 (1.63-3.55) | <0.001 | 1.56 (1.39-1.74) | <0.001 | 1.12 (1.09-1.16) | <0.001 |
|  |  |  |  |  |  |  |
| ANC: Antenatal Care, BMI: Body Mass Index, CI: Confidence Interval, OR: Odds Ratio, p: level of significance | | | | | | |

| **Table S4**. Association between iron-and-folic-acid (IFA) consumption and timing of neonatal mortality (day 0-1, day 2-6, and day 7-27) and neonatal mortality (day 0-27). | | | | | | | | |
| --- | --- | --- | --- | --- | --- | --- | --- | --- |
|  | **Neonatal mortality (day 0-1)** | | **Neonatal mortality (day 2-6)** | | **Neonatal mortality (day 7-27)** | | **Neonatal mortality (day 0-27)** | |
|  | OR (95% CI) | p | OR (95% CI) | p | OR (95% CI) | p | OR (95% CI) | p |
| **Anaemia status and IFA intake** |  |  |  |  |  |  |  |  |
| No IFA | **1.00 (referent)** |  | **1.00 (referent)** |  | **1.00 (referent)** |  | **1.00 (referent)** |  |
| <100 IFA | **0.89 (0.78-1.01)** | **0.079** | **0.92 (0.76-1.12)** | **0.431** | **0.74 (0.59-0.94)** | **0.015** | **0.87 (0.79-0.96)** | **0.006** |
| ≥100 IFA | **0.74 (0.63-0.88)** | **<0.001** | **0.85 (0.67-1.07)** | **0.171** | **0.62 (0.46-0.84)** | **0.002** | **0.75 (0.66-0.84)** | **<0.001** |
| **Current age-group of mother** |  |  |  |  |  |  |  |  |
| 15-19 | 1.00 (referent) |  | 1.00 (referent) |  | 1.00 (referent) |  | 1.00 (referent) |  |
| 20-29 | 0.61 (0.47-0.79) | <0.001 | 0.51 (0.36-0.71) | <0.001 | 0.30 (0.20-0.45) | <0.001 | 0.51 (0.42-0.61) | <0.001 |
| 30-39 | 0.55 (0.41-0.74) | <0.001 | 0.48 (0.32-0.72) | <0.001 | 0.25 (0.15-0.41) | <0.001 | 0.46 (0.37-0.56) | <0.001 |
| ≥40 | 0.72 (0.48-1.08) | 0.112 | 0.59 (0.33-1.07) | 0.081 | 0.21 (0.10-0.45) | <0.001 | 0.55 (0.40-0.75) | <0.001 |
| **Mother’s age at first birth** |  |  |  |  |  |  |  |  |
| <18 | 1.00 (referent) |  | 1.00 (referent) |  | 1.00 (referent) |  | 1.00 (referent) |  |
| ≥18 | 1.08 (0.91-1.28) | 0.372 | 1.36 (1.04-1.77) | 0.023 | 1.45 (1.04-2.00) | 0.026 | 1.21 (1.06-1.37) | 0.005 |
| **Education of mother** |  |  |  |  |  |  |  |  |
| No or incomplete primary | 1.00 (referent) |  | 1.00 (referent) |  | 1.00 (referent) |  | 1.00 (referent) |  |
| Primary or incomplete secondary | 0.89 (0.79-1.01) | 0.076 | 0.85 (0.70-1.01) | 0.071 | 1.00 (0.79-1.28) | 0.971 | 0.89 (0.81-0.98) | 0.023 |
| Secondary or higher | 0.74 (0.61-0.91) | 0.003 | 0.66 (0.50-0.87) | 0.004 | 0.71 (0.49-1.03) | 0.073 | 0.71 (0.61-0.82) | <0.001 |
| **Sex of child** |  |  |  |  |  |  |  |  |
| Male | 1.00 (referent) |  | 1.00 (referent) |  | 1.00 (referent) |  | 1.00 (referent) |  |
| Female | 0.90 (0.81-0.99) | 0.039 | 0.95 (0.82-1.10) | 0.487 | 0.99 (0.81-1.20) | 0.899 | 0.92 (0.85-1.00) | 0.048 |
| **Birth order** |  |  |  |  |  |  |  |  |
| 1 | 1.00 (referent) |  | 1.00 (referent) |  | 1.00 (referent) |  | 1.00 (referent) |  |
| 2 | 0.73 (0.63-0.84) | <0.001 | 0.68 (0.56-0.82) | <0.001 | 0.96 (0.74-1.25) | 0.773 | 0.74 (0.67-0.82) | <0.001 |
| 3 | 0.87 (0.74-1.03) | 0.108 | 0.67 (0.52-0.86) | 0.002 | 1.21 (0.88-1.66) | 0.244 | 0.85 (0.75-0.97) | 0.015 |
| 4 | 1.03 (0.84-1.28) | 0.754 | 1.00 (0.74-1.35) | 0.992 | 1.43 (0.96-2.15) | 0.081 | 1.08 (0.92-1.26) | 0.361 |
| ≥5 | 1.12 (0.89-1.42) | 0.337 | 0.96 (0.68-1.37) | 0.834 | 2.18 (1.42-3.34) | <0.001 | 1.21 (1.01-1.44) | 0.040 |
| **Place of residence** |  |  |  |  |  |  |  |  |
| Urban | 1.00 (referent) |  | 1.00 (referent) |  | 1.00 (referent) |  | 1.00 (referent) |  |
| Rural | 0.99 (0.86-1.14) | 0.873 | 1.10 (0.89-1.36) | 0.362 | 1.18 (0.90-1.55) | 0.235 | 1.05 (0.94-1.17) | 0.399 |
| **Social group** |  |  |  |  |  |  |  |  |
| Others | 1.00 (referent) |  | 1.00 (referent) |  | 1.00 (referent) |  | 1.00 (referent) |  |
| Scheduled castes | 1.35 (1.14-1.60) | 0.001 | 1.44 (1.12-1.85) | 0.005 | 1.21 (0.88-1.66) | 0.242 | 1.35 (1.19-1.54) | <0.001 |
| Scheduled tribes | 0.89 (0.73-1.09) | 0.270 | 1.09 (0.81-1.45) | 0.579 | 1.07 (0.75-1.54) | 0.695 | 0.97 (0.83-1.13) | 0.692 |
| Other Backward Classes | 1.00 (0.86-1.16) | 0.978 | 1.19 (0.95-1.49) | 0.131 | 0.92 (0.70-1.21) | 0.547 | 1.03 (0.92-1.16) | 0.591 |
| **Religion** |  |  |  |  |  |  |  |  |
| Hinduism | 1.00 (referent) |  | 1.00 (referent) |  | 1.00 (referent) |  | 1.00 (referent) |  |
| Islam | 1.20 (1.03-1.40) | 0.023 | 1.00 (0.79-1.27) | 0.969 | 1.11 (0.83-1.49) | 0.466 | 1.13 (1.01-1.28) | 0.040 |
| Christianity | 0.92 (0.70-1.22) | 0.576 | 0.48 (0.30-0.79) | 0.004 | 0.40 (0.21-0.77) | 0.006 | 0.70 (0.56-0.88) | 0.002 |
| Others | 1.07 (0.80-1.44) | 0.651 | 0.89 (0.57-1.38) | 0.598 | 0.83 (0.47-1.47) | 0.523 | 0.98 (0.78-1.22) | 0.827 |
| **Economic group** |  |  |  |  |  |  |  |  |
| Poorest | 1.00 (referent) |  | 1.00 (referent) |  | 1.00 (referent) |  | 1.00 (referent) |  |
| Poorer | 1.01 (0.87-1.17) | 0.913 | 1.11 (0.90-1.37) | 0.309 | 0.68 (0.51-0.91) | 0.008 | 0.98 (0.87-1.09) | 0.672 |
| Middle | 0.87 (0.74-1.03) | 0.113 | 0.92 (0.72-1.17) | 0.483 | 0.80 (0.59-1.08) | 0.146 | 0.87 (0.77-0.99) | 0.030 |
| Richer | 0.69 (0.57-0.85) | <0.001 | 0.73 (0.54-0.98) | 0.036 | 0.61 (0.42-0.89) | 0.011 | 0.69 (0.59-0.80) | <0.001 |
| Richest | 0.53 (0.41-0.68) | <0.001 | 0.75 (0.53-1.06) | 0.104 | 0.77 (0.50-1.18) | 0.232 | 0.62 (0.51-0.74) | <0.001 |
| **State of residence** |  |  |  |  |  |  |  |  |
| Non-high focus | 1.00 (referent) |  | 1.00 (referent) |  | 1.00 (referent) |  | 1.00 (referent) |  |
| High Focus | 1.52 (1.32-1.74) | <0.001 | 1.51 (1.24-1.83) | <0.001 | 1.47 (1.14-1.89) | 0.003 | 1.51 (1.37-1.68) | <0.001 |
| **Number of ANC visit** |  |  |  |  |  |  |  |  |
| ≥4 | 1.00 (referent) |  | 1.00 (referent) |  | 1.00 (referent) |  | 1.00 (referent) |  |
| <4 | 1.11 (0.98-1.24) | 0.090 | 1.05 (0.89-1.24) | 0.588 | 0.98 (0.79-1.21) | 0.834 | 1.07 (0.98-1.17) | 0.139 |
| **Received supplementary food from *Anganwadi* centre** |  |  |  |  |  |  |  |  |
| Yes | 1.00 (referent) |  | 1.00 (referent) |  | 1.00 (referent) |  | 1.00 (referent) |  |
| No | 1.29 (1.15-1.44) | <0.001 | 1.17 (1.00-1.38) | 0.052 | 1.44 (1.17-1.77) | 0.001 | 1.28 (1.18-1.39) | <0.001 |
| **Blood sample taken during ANC visit** |  |  |  |  |  |  |  |  |
| Yes | 1.00 (referent) |  | 1.00 (referent) |  | 1.00 (referent) |  | 1.00 (referent) |  |
| No | 0.87 (0.75-1.00) | 0.054 | 0.83 (0.67-1.03) | 0.093 | 0.87 (0.66-1.14) | 0.302 | 0.85 (0.76-0.96) | 0.006 |
| **Institutional delivery** |  |  |  |  |  |  |  |  |
| Yes | 1.00 (referent) |  | 1.00 (referent) |  | 1.00 (referent) |  | 1.00 (referent) |  |
| No | 1.11 (0.97-1.27) | 0.128 | 0.99 (0.81-1.22) | 0.953 | 1.10 (0.85-1.41) | 0.474 | 1.08 (0.97-1.20) | 0.155 |
| **BMI of mother** |  |  |  |  |  |  |  |  |
| Optimum | 1.00 (referent) |  | 1.00 (referent) |  | 1.00 (referent) |  | 1.00 (referent) |  |
| Underweight | 0.80 (0.70-0.91) | 0.001 | 0.80 (0.67-0.96) | 0.019 | 0.90 (0.71-1.16) | 0.421 | 0.81 (0.73-0.90) | <0.001 |
| Overweight and obesity | 1.26 (1.11-1.44) | <0.001 | 0.92 (0.76-1.12) | 0.405 | 1.37 (1.08-1.74) | 0.010 | 1.18 (1.07-1.30) | 0.001 |
|  |  |  |  |  |  |  |  |  |
| ANC: Antenatal Care, BMI: Body Mass Index, CI: Confidence Interval, OR: Odds Ratio, p: level of significance | | | | | | | | |


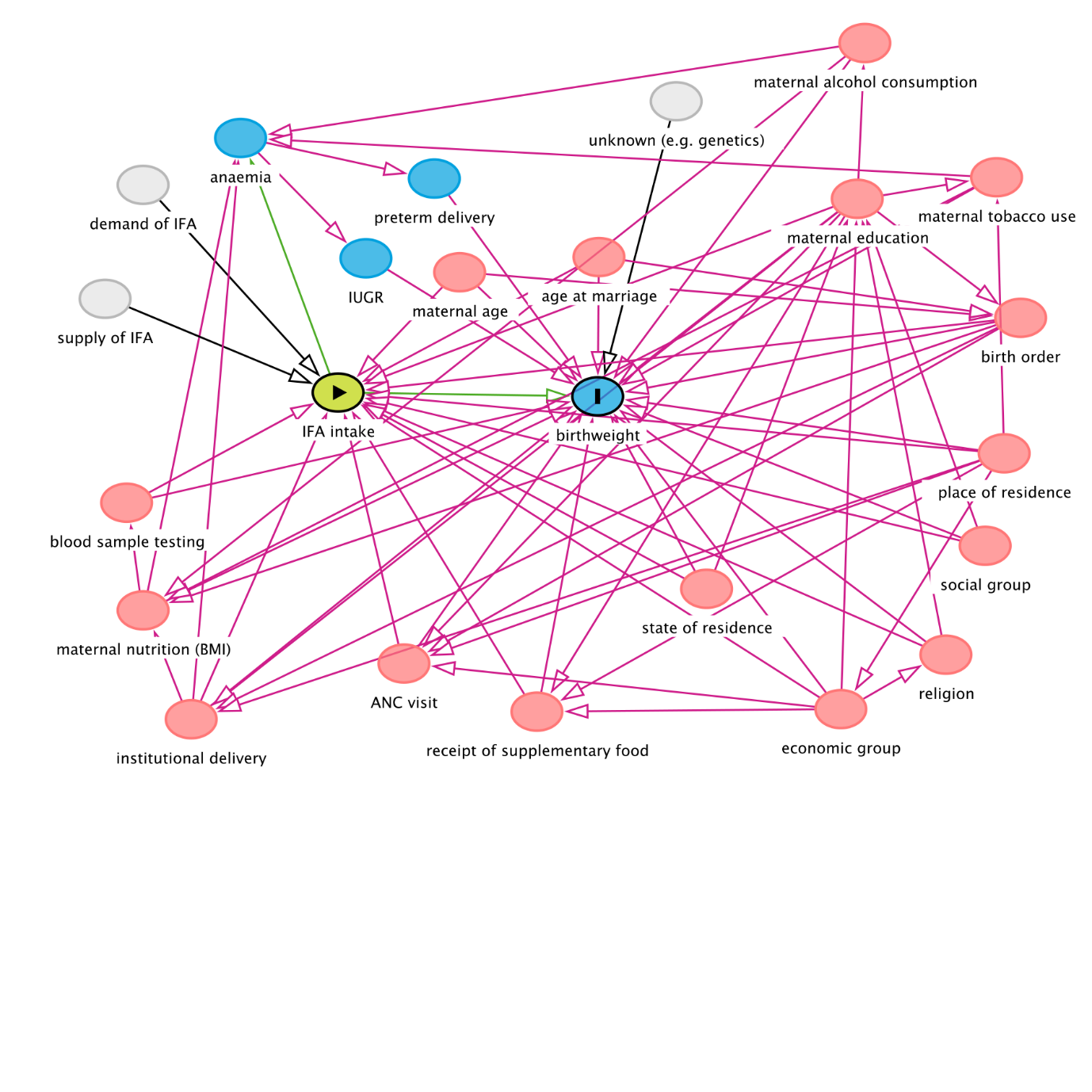


**Figure S1**. Directed Acyclic Graph (DAG) showing the causal pathways between exposure (IFA or iron-and-folic-acid tablet/ syrup intake during pregnancy) and outcome (birthweight) variable.

Sources of birthweight data, a potential confounder for the causal relationship between IFA intake and birthweight, is not shown separately.

ANC: antenatal care, BMI: Body Mass Index, IUGR: intrauterine growth restriction.

**Legend**

exposure,
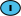
outcome,
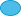
ancestor of outcome,
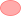
ancestor of exposure and outcome,


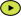


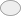
unobserved (latent),
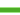
causal path,
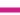
biasing path

Figure was developed using online browser - <http://www.dagitty.net/>

**Note**: A separate DAG on the causal pathways between IFA intake (exposure) and neonatal mortality (outcome) is not shown separately, as it is similar to Figure S1, except that sex of the child was identified as an additional confounder for the causal relationship between IFA intake and neonatal mortality.
